# 5-Bromo-2-hy­droxy­benzonitrile

**DOI:** 10.1107/S1600536812031716

**Published:** 2012-08-01

**Authors:** Scott Oh, Joseph M. Tanski

**Affiliations:** a Department of Chemistry, Vassar College, Poughkeepsie, NY 12604, USA

## Abstract

The title compound, C_7_H_4_BrNO, crystallizes with two mol­ecules in the asymmetric unit. The two molecules exhibit nearly linear C—C N nitrile bond angles of 179.1 (4) and 177.1 (4)°. In the crystal, the mol­ecules are linked into a one-dimensional hydrogen-bonded chain by inter­actions between the phenol H atom and the nitrile N atom [N⋯O = 2.805 (4) and 2.810 (4) Å].

## Related literature

For information on the synthesis of the title compound, see: Anwar & Hansen (2008 ▶); Bonnichon *et al.* (1999 ▶); Oberhauser (1997 ▶); Tamilselvan *et al.* (2009 ▶). For use as a synthetic reagent, see: Jiang *et al.* (2011 ▶); Tsuhako *et al.* (2012 ▶); Wetzel *et al.* (2011 ▶). For a related crystal structure, see: Beswick *et al.* (1996 ▶).
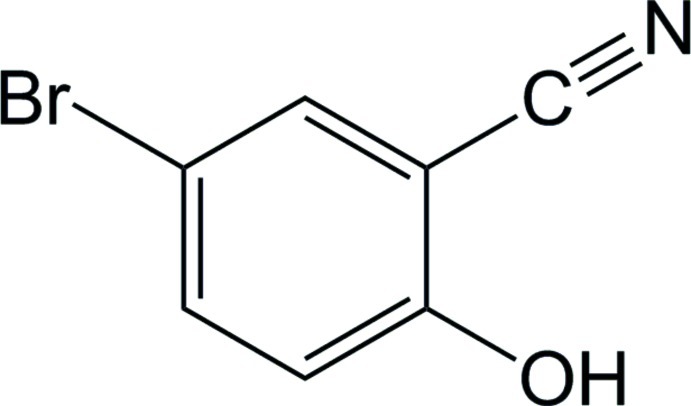


## Experimental

### 

#### Crystal data

C_7_H_4_BrNO
*M* *_r_* = 198.01
Triclinic, 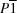

*a* = 3.8422 (3) Å
*b* = 8.5166 (7) Å
*c* = 21.6507 (18) Å
α = 97.074 (1)°
β = 91.991 (1)°
γ = 97.068 (1)°
*V* = 696.83 (10) Å^3^
*Z* = 4
Mo *K*α radiation
μ = 5.82 mm^−1^
*T* = 125 K
0.20 × 0.07 × 0.03 mm

#### Data collection

Bruker APEXII CCD diffractometer
Absorption correction: multi-scan (*SADABS*; Bruker, 2007 ▶) *T*_min_ = 0.389, *T*_max_ = 0.845
11040 measured reflections
4213 independent reflections
3254 reflections with *I* > 2σ(*I*)
*R*_int_ = 0.032

#### Refinement

*R*[*F* ^2^ > 2σ(*F* ^2^)] = 0.043
*wR*(*F* ^2^) = 0.100
*S* = 1.03
4213 reflections
187 parameters
2 restraints
H atoms treated by a mixture of independent and constrained refinement
Δρ_max_ = 1.67 e Å^−3^
Δρ_min_ = −0.57 e Å^−3^

### 

Data collection: *APEX2* (Bruker, 2007 ▶); cell refinement: *SAINT* (Bruker, 2007 ▶); data reduction: *SAINT*; program(s) used to solve structure: *SHELXS97* (Sheldrick, 2008 ▶); program(s) used to refine structure: *SHELXL97* (Sheldrick, 2008 ▶); molecular graphics: *SHELXTL* (Sheldrick, 2008 ▶); software used to prepare material for publication: *SHELXTL*.

## Supplementary Material

Crystal structure: contains datablock(s) I, global. DOI: 10.1107/S1600536812031716/rk2372sup1.cif

Structure factors: contains datablock(s) I. DOI: 10.1107/S1600536812031716/rk2372Isup2.hkl

Supplementary material file. DOI: 10.1107/S1600536812031716/rk2372Isup3.cml

Additional supplementary materials:  crystallographic information; 3D view; checkCIF report

## Figures and Tables

**Table 1:** Hydrogen-bond geometry (Å, °)

*D*—H⋯*A*	*D*—H	H⋯*A*	*D*⋯*A*	*D*—H⋯*A*
O1—H1⋯N2	0.83 (2)	1.98 (2)	2.805 (4)	170 (5)
O2—H2⋯N1^i^	0.84 (2)	1.98 (2)	2.810 (4)	175 (5)

Symmetry code: (i) 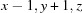.

**Fig. 1.**
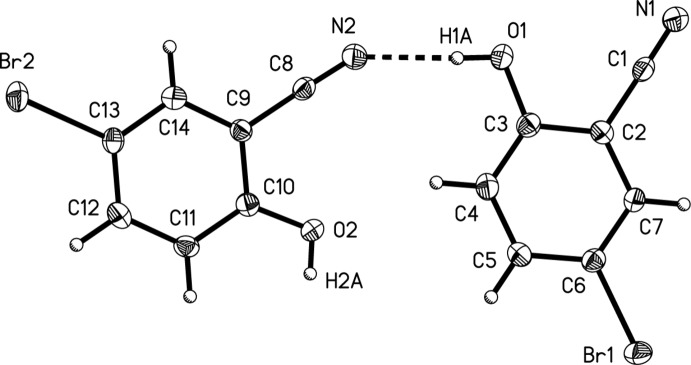
A view of the two independent molecules of the title compound with the atom numbering scheme. Displacement ellipsoids are shown at the 50% probability level. H atoms are presented as a small spheres of arbitrary radius. One of the hydrogen bonds is drawn with a dashed line.

**Fig. 2.**
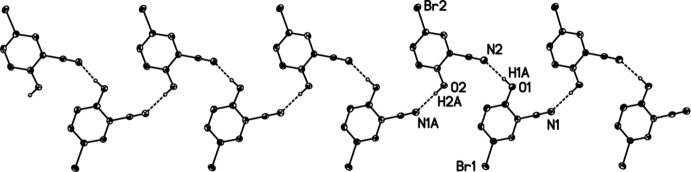
A view of the one-dimensional hydrogen bonding chain. H atoms not involved in H-bonds are omitted for clarity.

## supplementary crystallographic information

### Comment

The title compound, 5-bromo-2-hydroxybenzonitrile, may be prepared by a variety of methods, including bromination of *o*-cyanophenol (Oberhauser, 1997), additon of nitrile to *p*-bromophenol (Anwar & Hansen, 2008), cobalt(II) catalyzed conversion of 5-bromo-2-hydroxyaldoxime to the nitrile (Tamilselvan *et al.*, 2009), and photochemically from 5-chloro-2-hydroxybenzonitrile in the presence of bromide ions (Bonnichon *et al.*, 1999). The 5-bromo-2-hydroxybenzonitrile is used as a synthetic reagent in the synthesis of biologically active compounds such as potential antiretroviral drugs (Jiang *et al.*, 2011), cancer therapies (Tsuhako *et al.*, 2012), and osteoporosis treatments (Wetzel *et al.*, 2011).

The asymmetric unit contains two uniqe molecules of the title compound (Fig. 1) which are hydrogen bonded into an infinite one-dimensional chain (Fig. 2). The phenoxy group acts as the hydrogen donor and the nitrile group as the acceptor, with O···N distances of 2.805 (4)Å and 2.810 (4)Å, and O–H···N angles of 170 (5)° and 175 (5)°. The metrical parameters are similar to those found in the structure of *o*-cyanonitrile, which also crystallizes with two molecules in the asymmetric unit, and exhibts O···N distances of 2.795 (2)Å and 2.798 (2)Å, and O–H···N angles of 173 (2)° and 172 (2)° (Beswick *et al.*, 1996). As in the structure of *o*-cyanonitrile, the molecules of the title compound are nearly planar, with a root mean square deviations from the plane of all atoms, excluding the aryl H atoms, of 0.0334Å and 0.2747Å. In each molecule in the asymmetric unit, the greatest deviation from the plane is the phenolic hydrogen atom, presumably to maximize the hydrogen bonding interaction between neighboring molecules, which make a dihedral angle between them of 12.6 (5)°.

### Experimental

Crystalline 5-bromo-2-hydroxybenzonitrile was purchased from Aldrich Chemical Company, USA, and was recrystallized from chloroform.

### Refinement

Hydrogen atoms based on carbon were included in calculated positions and refined using a riding model at C–H = 0.95Å and *U*_iso_(H) = 1.2*U*_eq_(C_aryl_). Hydrogen atoms based on oxygen were refined semifreely with the help of a distance restraint O–H = 0.84Å, and *U*_iso_(H) = 1.5*U*_eq_(O).

### Crystal data

**Table:**

C_7_H_4_BrNO	*Z* = 4
*M**_r_* = 198.01	*F*(000) = 384
Triclinic, *P*1	*D*_x_ = 1.888 Mg m^−^^3^
Hall symbol: -P 1	Mo *K*α radiation, λ = 0.71073 Å
*a* = 3.8422 (3) Å	Cell parameters from 5206 reflections
*b* = 8.5166 (7) Å	θ = 2.5–30.5°
*c* = 21.6507 (18) Å	µ = 5.82 mm^−^^1^
α = 97.074 (1)°	*T* = 125 K
β = 91.991 (1)°	Needle, colourless
γ = 97.068 (1)°	0.20 × 0.07 × 0.03 mm
*V* = 696.83 (10) Å^3^	

### Data collection

**Table:**

Bruker APEXII CCD diffractometer	4213 independent reflections
Radiation source: fine-focus sealed tube	3254 reflections with *I* > 2σ(*I*)
Graphite monochromator	*R*_int_ = 0.032
φ and ω scans	θ_max_ = 30.5°, θ_min_ = 1.9°
Absorption correction: multi-scan (*SADABS*; Bruker, 2007)	*h* = −5→5
*T*_min_ = 0.389, *T*_max_ = 0.845	*k* = −12→12
11040 measured reflections	*l* = −30→30

### Refinement

**Table:**

Refinement on *F*^2^	Primary atom site location: structure-invariant direct methods
Least-squares matrix: full	Secondary atom site location: difference Fourier map
*R*[*F*^2^ > 2σ(*F*^2^)] = 0.043	Hydrogen site location: inferred from neighbouring sites
*wR*(*F*^2^) = 0.100	H atoms treated by a mixture of independent and constrained refinement
*S* = 1.03	*w* = 1/[σ^2^(*F*_o_^2^) + (0.050*P*)^2^ + 0.6935*P*] where *P* = (*F*_o_^2^ + 2*F*_c_^2^)/3
4213 reflections	(Δ/σ)_max_ = 0.001
187 parameters	Δρ_max_ = 1.67 e Å^−^^3^
2 restraints	Δρ_min_ = −0.57 e Å^−^^3^

### Special details

**Table:**

Geometry. All s.u.'s (except the s.u. in the dihedral angle between two l.s. planes) are estimated using the full covariance matrix. The cell s.u.'s are taken into account individually in the estimation of s.u.'s in distances, angles and torsion angles; correlations between s.u.'s in cell parameters are only used when they are defined by crystal symmetry. An approximate (isotropic) treatment of cell s.u.'s is used for estimating s.u.'s involving l.s. planes.
Refinement. Refinement of *F*^2^ against ALL reflections. The weighted *R*-factor *wR* and goodness of fit *S* are based on *F*^2^, conventional *R*-factors *R* are based on *F*, with *F* set to zero for negative *F*^2^. The threshold expression of *F*^2^ > σ(*F*^2^) is used only for calculating *R*-factors(gt) *etc*. and is not relevant to the choice of reflections for refinement. *R*-factors based on *F*^2^ are statistically about twice as large as those based on *F*, and *R*-factors based on ALL data will be even larger.

### Fractional atomic coordinates and isotropic or equivalent isotropic displacement parameters (Å^2^)

**Table:**

	*x*	*y*	*z*	*U*_iso_*/*U*_eq_	
Br1	0.18318 (9)	0.28671 (4)	0.512507 (15)	0.02561 (10)	
Br2	0.29449 (9)	0.79771 (4)	0.004330 (16)	0.02483 (10)	
O1	0.5299 (7)	0.0480 (3)	0.25051 (11)	0.0272 (5)	
H1	0.477 (12)	0.107 (5)	0.2245 (17)	0.041*	
O2	−0.0783 (7)	0.5153 (3)	0.23932 (11)	0.0256 (5)	
H2	−0.155 (11)	0.581 (4)	0.2658 (16)	0.038*	
N1	0.6255 (9)	−0.2737 (4)	0.32638 (14)	0.0297 (7)	
N2	0.2873 (9)	0.2144 (4)	0.15707 (14)	0.0284 (7)	
C1	0.5484 (9)	−0.1490 (4)	0.33933 (15)	0.0219 (6)	
C2	0.4527 (8)	0.0091 (4)	0.35481 (15)	0.0198 (6)	
C3	0.4427 (9)	0.1083 (4)	0.30815 (15)	0.0205 (6)	
C4	0.3500 (9)	0.2610 (4)	0.32282 (16)	0.0243 (7)	
H4A	0.3393	0.3289	0.2913	0.029*	
C5	0.2735 (9)	0.3141 (4)	0.38330 (16)	0.0224 (6)	
H5A	0.2114	0.4185	0.3933	0.027*	
C6	0.2875 (8)	0.2141 (4)	0.42964 (15)	0.0200 (6)	
C7	0.3737 (8)	0.0620 (4)	0.41583 (14)	0.0192 (6)	
H7A	0.3793	−0.0061	0.4473	0.023*	
C8	0.2157 (9)	0.3355 (4)	0.14778 (15)	0.0213 (6)	
C9	0.1384 (8)	0.4920 (4)	0.13853 (15)	0.0188 (6)	
C10	−0.0049 (8)	0.5841 (4)	0.18745 (15)	0.0196 (6)	
C11	−0.0606 (9)	0.7398 (4)	0.17991 (15)	0.0211 (6)	
H11A	−0.1568	0.8038	0.2124	0.025*	
C12	0.0238 (9)	0.8011 (4)	0.12526 (16)	0.0218 (6)	
H12A	−0.0129	0.9073	0.1205	0.026*	
C13	0.1624 (8)	0.7076 (4)	0.07722 (15)	0.0190 (6)	
C14	0.2219 (8)	0.5542 (4)	0.08324 (15)	0.0196 (6)	
H14A	0.3182	0.4913	0.0504	0.023*	

### Atomic displacement parameters (Å^2^)

**Table:**

	*U*^11^	*U*^22^	*U*^33^	*U*^12^	*U*^13^	*U*^23^
Br1	0.02688 (19)	0.02853 (19)	0.02188 (17)	0.00764 (14)	0.00549 (13)	−0.00014 (13)
Br2	0.02437 (18)	0.02336 (17)	0.02838 (18)	0.00123 (13)	0.00333 (13)	0.01102 (13)
O1	0.0410 (15)	0.0240 (13)	0.0186 (11)	0.0098 (11)	0.0007 (10)	0.0047 (9)
O2	0.0380 (14)	0.0226 (12)	0.0173 (11)	0.0072 (11)	0.0050 (10)	0.0026 (9)
N1	0.0419 (19)	0.0259 (16)	0.0231 (15)	0.0104 (13)	0.0048 (13)	0.0035 (12)
N2	0.0417 (18)	0.0210 (15)	0.0223 (14)	0.0063 (13)	−0.0009 (13)	0.0004 (11)
C1	0.0268 (17)	0.0235 (17)	0.0159 (14)	0.0034 (13)	0.0015 (12)	0.0046 (12)
C2	0.0197 (15)	0.0184 (15)	0.0215 (15)	0.0034 (12)	0.0004 (12)	0.0024 (12)
C3	0.0193 (15)	0.0231 (16)	0.0184 (15)	0.0016 (12)	−0.0005 (12)	0.0018 (12)
C4	0.0289 (18)	0.0220 (16)	0.0230 (16)	0.0050 (13)	−0.0003 (13)	0.0058 (13)
C5	0.0246 (16)	0.0196 (15)	0.0235 (16)	0.0057 (12)	0.0008 (13)	0.0025 (12)
C6	0.0177 (15)	0.0242 (16)	0.0182 (15)	0.0036 (12)	0.0014 (12)	0.0023 (12)
C7	0.0204 (15)	0.0205 (15)	0.0170 (14)	0.0028 (12)	−0.0005 (12)	0.0040 (12)
C8	0.0268 (16)	0.0213 (16)	0.0154 (14)	0.0021 (13)	0.0010 (12)	0.0017 (12)
C9	0.0207 (15)	0.0170 (14)	0.0185 (14)	0.0031 (12)	−0.0004 (12)	0.0016 (11)
C10	0.0199 (15)	0.0179 (15)	0.0198 (15)	0.0002 (11)	−0.0021 (12)	0.0009 (12)
C11	0.0235 (16)	0.0183 (15)	0.0201 (15)	0.0026 (12)	−0.0008 (12)	−0.0021 (12)
C12	0.0218 (16)	0.0170 (15)	0.0260 (16)	0.0023 (12)	−0.0043 (13)	0.0025 (12)
C13	0.0175 (14)	0.0184 (15)	0.0209 (15)	−0.0009 (11)	0.0002 (12)	0.0051 (12)
C14	0.0167 (14)	0.0196 (15)	0.0214 (15)	0.0005 (11)	0.0003 (12)	0.0004 (12)

### Geometric parameters (Å, º)

**Table:**

Br1—C6	1.897 (3)	C5—C6	1.397 (4)
Br2—C13	1.896 (3)	C5—H5A	0.9500
O1—C3	1.359 (4)	C6—C7	1.376 (4)
O1—H1	0.834 (19)	C7—H7A	0.9500
O2—C10	1.352 (4)	C8—C9	1.436 (4)
O2—H2	0.836 (19)	C9—C14	1.399 (4)
N1—C1	1.142 (4)	C9—C10	1.408 (4)
N2—C8	1.139 (4)	C10—C11	1.396 (4)
C1—C2	1.442 (4)	C11—C12	1.384 (5)
C2—C3	1.397 (4)	C11—H11A	0.9500
C2—C7	1.398 (4)	C12—C13	1.392 (5)
C3—C4	1.393 (5)	C12—H12A	0.9500
C4—C5	1.385 (5)	C13—C14	1.375 (4)
C4—H4A	0.9500	C14—H14A	0.9500
			
C3—O1—H1	110 (3)	C2—C7—H7A	120.4
C10—O2—H2	110 (3)	N2—C8—C9	177.1 (4)
N1—C1—C2	179.1 (4)	C14—C9—C10	120.9 (3)
C3—C2—C7	120.8 (3)	C14—C9—C8	120.4 (3)
C3—C2—C1	119.0 (3)	C10—C9—C8	118.6 (3)
C7—C2—C1	120.2 (3)	O2—C10—C11	124.1 (3)
O1—C3—C4	124.0 (3)	O2—C10—C9	117.3 (3)
O1—C3—C2	116.7 (3)	C11—C10—C9	118.7 (3)
C4—C3—C2	119.3 (3)	C12—C11—C10	120.2 (3)
C5—C4—C3	120.1 (3)	C12—C11—H11A	119.9
C5—C4—H4A	119.9	C10—C11—H11A	119.9
C3—C4—H4A	119.9	C11—C12—C13	120.2 (3)
C4—C5—C6	120.0 (3)	C11—C12—H12A	119.9
C4—C5—H5A	120.0	C13—C12—H12A	119.9
C6—C5—H5A	120.0	C14—C13—C12	121.0 (3)
C7—C6—C5	120.7 (3)	C14—C13—Br2	119.6 (2)
C7—C6—Br1	119.2 (2)	C12—C13—Br2	119.3 (2)
C5—C6—Br1	120.1 (2)	C13—C14—C9	118.9 (3)
C6—C7—C2	119.1 (3)	C13—C14—H14A	120.5
C6—C7—H7A	120.4	C9—C14—H14A	120.5
			
C7—C2—C3—O1	178.8 (3)	C14—C9—C10—O2	−180.0 (3)
C1—C2—C3—O1	−0.8 (5)	C8—C9—C10—O2	−3.4 (4)
C7—C2—C3—C4	−0.5 (5)	C14—C9—C10—C11	−0.4 (5)
C1—C2—C3—C4	179.9 (3)	C8—C9—C10—C11	176.2 (3)
O1—C3—C4—C5	−178.4 (3)	O2—C10—C11—C12	179.7 (3)
C2—C3—C4—C5	0.9 (5)	C9—C10—C11—C12	0.1 (5)
C3—C4—C5—C6	−0.3 (5)	C10—C11—C12—C13	0.5 (5)
C4—C5—C6—C7	−0.6 (5)	C11—C12—C13—C14	−0.8 (5)
C4—C5—C6—Br1	179.9 (3)	C11—C12—C13—Br2	−177.3 (2)
C5—C6—C7—C2	1.0 (5)	C12—C13—C14—C9	0.5 (5)
Br1—C6—C7—C2	−179.6 (2)	Br2—C13—C14—C9	177.0 (2)
C3—C2—C7—C6	−0.4 (5)	C10—C9—C14—C13	0.1 (5)
C1—C2—C7—C6	179.2 (3)	C8—C9—C14—C13	−176.4 (3)

### Hydrogen-bond geometry (Å, º)

**Table:**

*D*—H···*A*	*D*—H	H···*A*	*D*···*A*	*D*—H···*A*
O1—H1···N2	0.83 (2)	1.98 (2)	2.805 (4)	170 (5)
O2—H2···N1^i^	0.84 (2)	1.98 (2)	2.810 (4)	175 (5)

Symmetry code: (i) *x*−1, *y*+1, *z*.
